# Role of selected salivary inflammatory cytokines in the diagnosis and prognosis of oral squamous cell carcinoma. A Systematic Review and Meta-analysis

**DOI:** 10.4317/medoral.25889

**Published:** 2023-04-26

**Authors:** Eloy Benito-Ramal, Sonia Egido-Moreno, Beatriz González-Navarro, Enric Jané-Salas, Xavier Roselló-Llabrés, José López-López

**Affiliations:** 1 Dentistry Grade Student. University Campus of Bellvitge, University of Barcelona, Barcelona, Spain.; 2 Department of Odontoestomatology, Faculty of Medicine and Health Sciences, School of Dentistry, University Campus of Bellvitge, University of Barcelona, Barcelona, Spain.; 3 Oral Health and Masticatory System Group, IDIBELL (Bellvitge Biomedical Research Institute), University of Barcelona, Barcelona, Spain; 4 Faculty Director and Head of Service of the Medical-Surgical Area of Dentistry Hospital, University of Barcelona, Barcelona, Spain

## Abstract

**Background:**

Oral squamous cell carcinoma (OSCC) is gradually increasing its incidence in our society. Unfortunately, this entity is diagnosed at an advanced stage in most patients, a fact that implies greater difficulty in its treatment and a worse prognosis. This systematic review aims to assess whether the cytokines IL-6, IL-8 and TNF-α are potential salivary biomarkers that allow early diagnosis of cancer.

**Material and Methods:**

An electronic search was performed in three databases (Pubmed, Scopus and Web of Science). We used the following keywords: "salivary cytokines", "saliva cytokines", "salivary interleukins", "biomarkers", "oral squamous cell carcinoma" and "diagnosis", combined with the Boolean operators "AND" and "OR".

**Results:**

128 publications were found and finally 23 articles were included in the review and 15 in the meta-analysis. It has been observed that the majority of OSCC patients express higher salivary concentrations of IL-6, IL-8 and TNF-α compared to the control (CL) and premalignant lesion (OPML) groups. It has also been observed that the different premalignant lesions do not have statistically significant differences in the salivary concentration of the cytokines, and on the other hand, differences have been observed between the different TNM stages. The meta-analysis has shown that the difference in concentration of IL-6, IL-8 and TNF-α is statistically significant between the CL group and the OSCC, and also between the CL group and OPML.

**Conclusions:**

There is sufficient evidence to affirm that IL-6, IL-8 and TNF-α are useful salivary cytokines in the early diagnosis and prognosis of OSCC. Although future studies are necessary to establish greater reliability of these biomarkers and thus be able to develop a valid diagnostic test.

** Key words:**Salivary cytokines, IL-6, IL-8, TNF-α, oral squamous cell carcinoma, diagnosis.

## Introduction

Head and neck cancer is the sixth most frequent malignant tumor in the world, with a high incidence in countries such as India or Southeast Asia. It is recognized that more than 90% are oral cavity squamous cell carcinomas (OSCC) (1,2). On many occasions, the presence of oral premalignant (or potentially malignant) lesions (OPML) in the oral mucosa warns of the future malignancy of the process (3). The diagnosis of OSCC is based on a clinical diagnosis, based on visual inspection and the ability to detect alterations in the oral mucosa, followed by a biopsy of the suspicious tissue and its histopathological analysis (4). Unfortunately, many cancers of the oral cavity and pharynx are diagnosed at an advanced stage, that is, when an extensive primary tumor, lymph node involvement, and/or metastasis are observed (5,6). It is for this reason that there are studies where new diagnostic methods have been investigated in order to detect OSCC early, establish effective treatments and improve the prognosis and survival rate of the patient (5,7,8).

In recent years it has been observed that, in the same way as a tissue biopsy or a blood test, saliva could be a medium that contains abundant molecular information capable of detecting different pathologies, not only local but also systemic. In part, this is due to the close relationship that saliva has with the oral mucosa and the fact that it contains molecules, cells and proteins that have been transported from the circulatory system to the salivary glands, as well as crevicular fluid and transudates from the mouth mucosa contributes to its composition. In this way, the analysis of oral fluids could constitute a non-invasive, safe, and economical alternative in controlling the risk of developing OSCC, in its diagnosis and in its postoperative assessment (5,7).

Thus, saliva presents different biomarkers that could inform about the patient's condition. Biomarkers act as indicators of normality or pathological processes. Therefore, they could provide information for the diagnosis and prognosis of the disease. The fact that saliva maintains direct contact with oral cancer lesions makes it a highly sensitive diagnostic tool. In fact, many genomic (DNA, RNA, mRNA) and proteomic (cytokines, chemokines, interferons, interleukins [IL], growth factors, proangiogenic factors, etc.) biomarkers have already been identified, among other molecules (5,7,8).

The role of proinflammatory cytokines in the human body is extensive. The inflammatory and immune response is known to play an important role in carcinogenesis, where cytokines control immune activation and cell proliferation, survival, and migration (9,10). In addition, many have a pro-angiogenic effect, favoring the growth of blood vessels in the tumor and, in this way, perpetuating its permanence and progression. In this way, it is reflected how they are involved in the processes of initiation, growth, invasion, and metastasis of cancer (10).

A multitude of studies have shown the highest concentration of salivary cytokines in patients with OSCC, thus supporting the idea that cytokines are potential diagnostic markers of cancer (5,7). However, those cytokines with real diagnostic potential for OSCC must be specified, as well as those capable of differentiating OSCC from OPML or other inflammatory pathologies.

The aim of this systematic review is to confirm the proposed hypothesis: The proinflammatory cytokines IL-6, IL-8 and tumor necrosis factor alpha (TNF-α) are reliable salivary biomarkers of OSCC, and their analysis allows differentiation between healthy patients, patients with OPML and patients with OSCC.

## Material and Methods

This systematic review and meta-analysis was carried out based on the PRISMA (Preferred Reporting Items for Systematic Reviews and Meta-Analyses) criteria (11). In order to carry out the review, a PICO question (Population, Intervention, Comparison and Outcome) has been prepared: Population: Patients with oral squamous cell carcinoma (OSCC) and in patients with potentially malignant lesions in the oral cavity (OPML). Intervention: Diagnostic test: analysis of the concentration of the proinflammatory cytokines IL-6, IL-8 and TNF-α present in saliva. Comparison: Analysis of the concentration of proinflammatory cytokines in control groups (CL). Results: IL-6, IL-8 and TNF-α are reliable salivary biomarkers with diagnostic capacity for OSCC and high discriminatory power of various premalignant lesions and the different stages of OSCC (S1-S4).

As a result of this research question, a search was carried out in the MEDLINE (PubMed), Web of Science (WOS) and Scopus databases in July and August 2022, considering all those publications between 1999 and 2022. The following keywords have been used in the respective search engines: “salivary cytokines”, “saliva cytokines”, “salivary interleukins”, “biomarkers”, “oral squamous cell carcinoma” and “diagnosis”, combined with the Boolean operators “AND” and “OR”.

First, all those duplicate articles have been removed. Subsequently, those showing a title and an abstract of interest have been selected, and the resulting records have been evaluated for full text eligibility. Finally, a manual search has been carried out through the Google Scholar database.

The studies included in the qualitative synthesis have been selected based on the following inclusion criteria: I.-Study design: Observational studies (OE) (cases and controls and comparative studies) conducted on human subjects. II.-Type of participants: Patients diagnosed with OSCC, patients with OPML and healthy participants (absence of any pathology that could alter the results) (CL). III.-Intervention: In all groups, the concentration of salivary cytokines (IL-6, IL-8 and TNF-α) in their protein form must be determined. IV.-Applied technique: Salivary cytokines must be measured by ELISA (Enzyme-linked immunosorbent assay). V.-Research question/study objective: Comparison of the levels of these salivary cytokines between the OSCC, OPML and CL groups, and determine if these correspond to reliable salivary biomarkers of oral cancer. There are no restrictions regarding the year of publication.

On the contrary, the exclusion criteria applied have been the following: I.-Study design: review articles, case report, notes, studies on non-human subjects, studies in a language other than English, books and documents. II.-Type of patients: patients with cancer other than OSCC or in patients undergoing OSCC treatment. III.-Intervention: analysis of other fluids or tissues (blood, mucosal biopsy, etc.) or analysis of other biomolecules (different cytokines, different molecules or cytokines in non-protein form). IV.-Applied technique: Cytokine analysis techniques other than ELISA. V.-Research question/objective of the study: other purposes to evaluate the diagnostic capacity of proinflammatory cytokines.

- Statistical analysis

The Review Manager 5.4 program was used as a tool to analyse the data, previously recorded in an Excel Table. Forest Plots were performed to graphically represent the difference between IL-6, IL-8, and TNF-α concentrations in the COCE and OPML groups compared to the CL group, with a 95% confidence interval (CI). For the significance level, the *p-value* (p)=0.05 was used. Heterogeneity was assessed using the I2 test.

## Results

**Figure 1:**
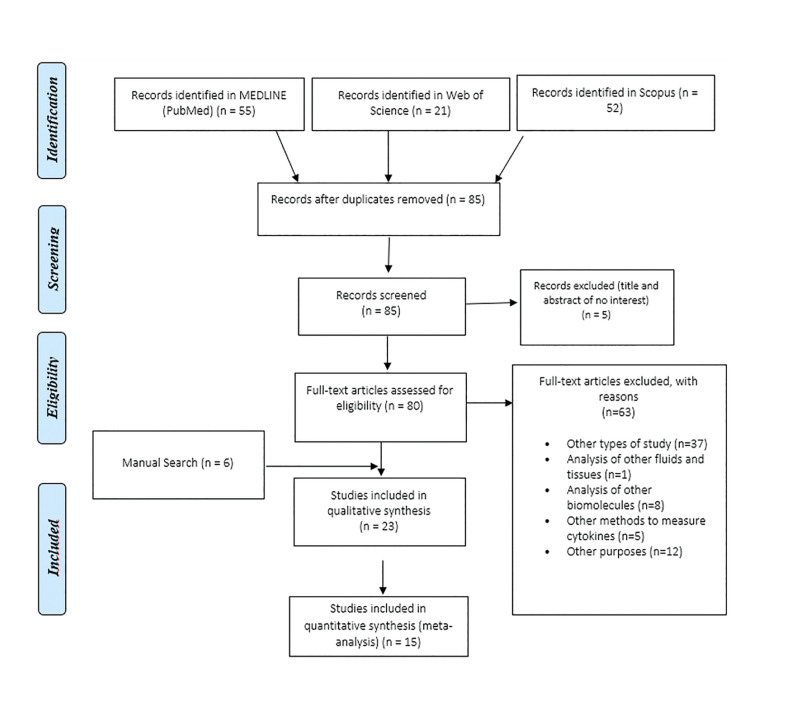
Study selection process summarized in the PRISMA flowchart.

In the initial electronic search, 128 articles were found. Duplicates were then excluded, leaving a total of 85. After reading the titles and abstracts, 80 articles were chosen for full text evaluation. Sixty-three studies were excluded by applying the different eligibility criteria. In addition, through a manual search, 6 records were identified in Google Scholar (12-15) that were evaluated and included based on the eligibility criteria. Finally, 23 publications were included in the systematic review (12-34) and the qualitative synthesis of the results has been carried out (Fig. 1).

The main characteristics of the included studies are shown in Table 1. The Newcastle Ottawa Scale (NOS) was used to assess the validity and methodological quality of the observational studies (Table 2).

**Table 1:**
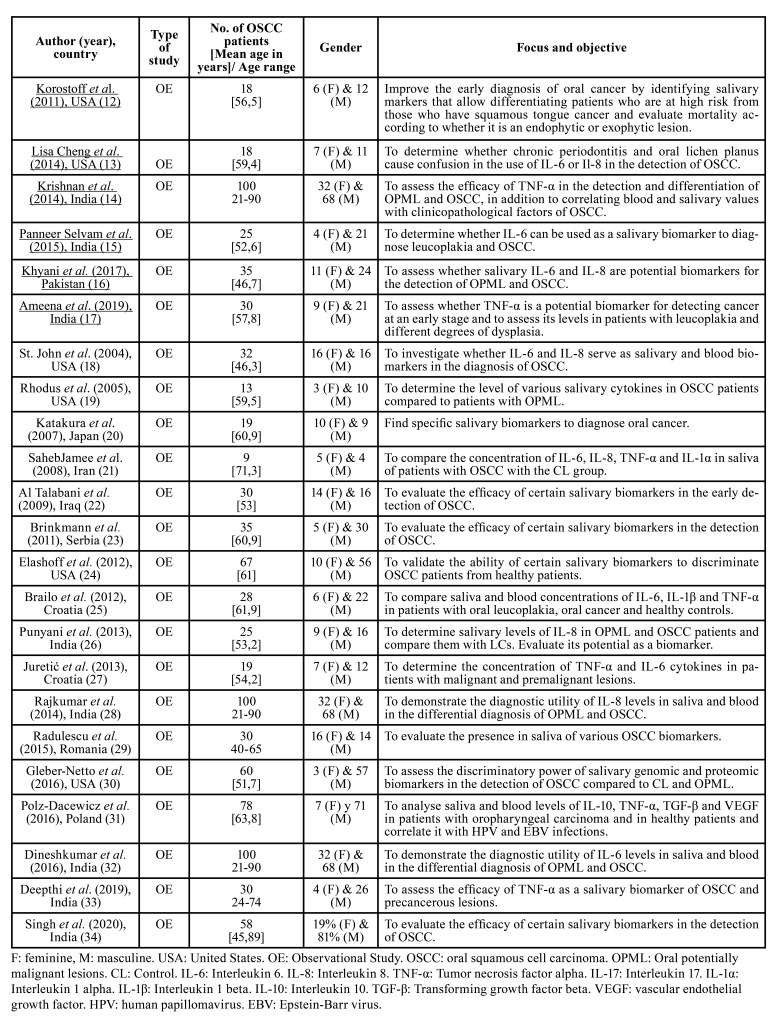
Description of the studies included in the systematic review.

**Table 2:**
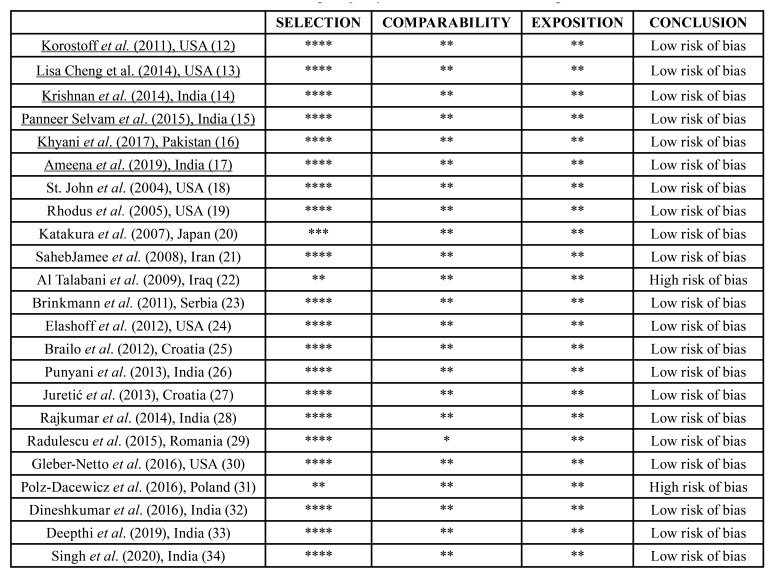
Assessment of the risk of bias and the methodological quality of the included studies according to the Newcastle-Ottawa scale.

The selected studies have been published between 2004 and 2020 and come from 10 different countries. Each of the included studies analyses the salivary concentration of IL-6, IL-8 and/or TNF-α in the CL and OSCC groups; and 14 of the 23 studies (14-16,17,19,25,30,32-34) include a third group of patients with OPML.

The OSCC sample size varies between 9 and 100 patients. The percentage of men and women is 72.89% and 27.11%, respectively. The total number of OSCC patients investigated is 959. The mean age of OSCC patients was 57 ± 5.67. However, some publications (14,28,29,32,33) did not provide the value of the mean age of their patients, but rather the range. Among the population groups studied in the 23 selected studies, the CL group has collected the most patients (*n*=997), followed by the OSCC group (*n*=959) and finally the OPML group (*n*=659). IL-6 has been investigated in 376 OSCC patients, IL-8 in 519 and TNF-α in 325. The frequency of appearance of IL-6 in the studies analysed is 56.52%, and that of IL- 8 and TNF-α is 60.87% and 39.13%, respectively (Table 3). The main results of the included studies are described below:

**Table 3:**
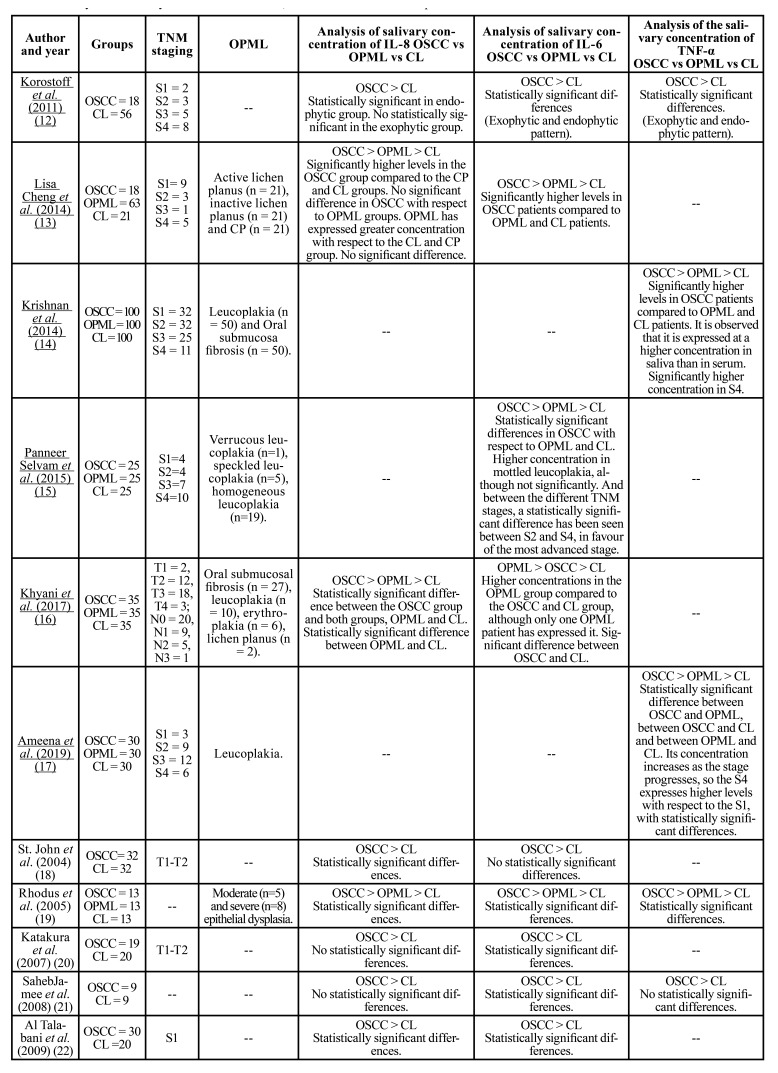
Analysis of salivary concentration of IL-6, IL-8 and TNF-α in oral squamous cell carcinoma.

**Table 3 cont.**
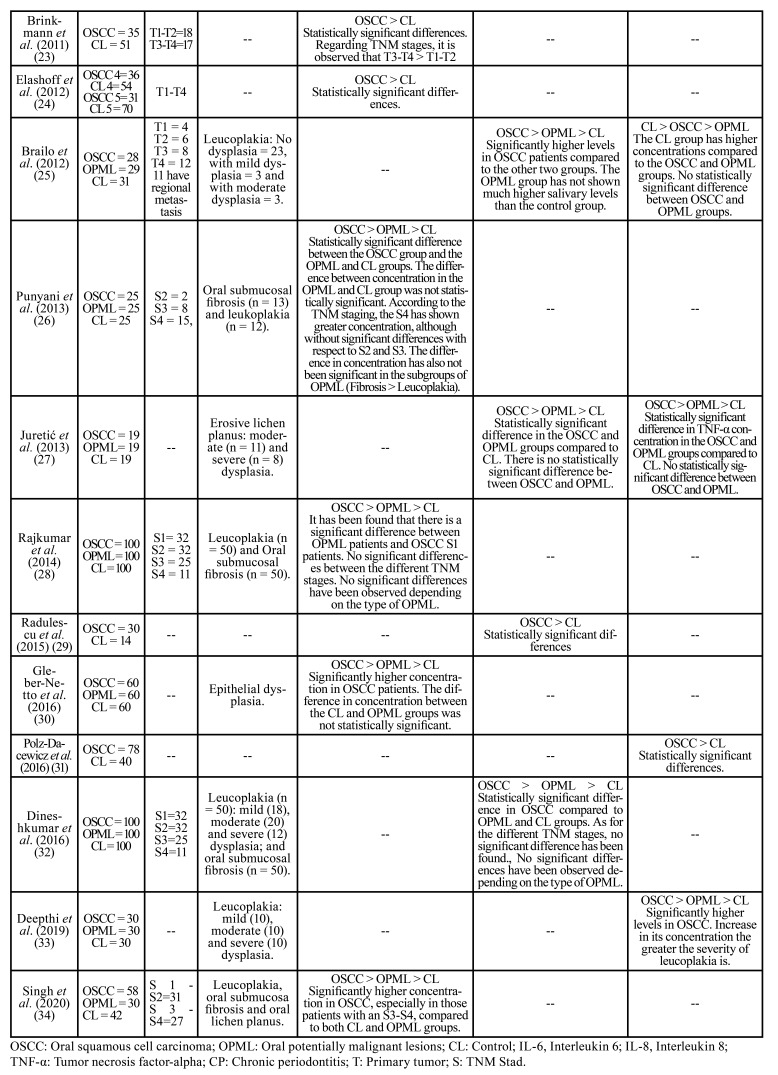
Analysis of salivary concentration of IL-6, IL-8 and TNF-α in oral squamous cell carcinoma.

- IL-6

IL-6 has been evaluated in 13 publications (12,13,15,16,18-22,25,27,29,32) (Table 3). It has been seen that IL-6 is found in saliva at higher concentrations in OSCC patients compared to the OPML and CL groups, except in one [37], where a single OPML patient has expressed IL-6 and has done so at a higher concentration than OSCC and CL patients. In 12 (12,13,15,16,19-22,25,27,29,32) of the 13 articles it is stated that the concentration of IL-6 is significantly higher in the OSCC group compared to the CL group. 6 of the studies (12,18,20-22,29) have only compared OSCC patients with CL patients, while 7 (13,15,16,19,25,27,32) have also compared salivary IL-6 levels in OSCC patients with OPML patients.

OPML have been leucoplakia, erythroplakia, erosive lichen planus, and oral submucosal fibrosis, with epithelial dysplasia (mild, moderate, and severe). In the study by Dineshkumar *et al*. (32), no significant differences have been observed in the salivary levels of IL-6 between the different premalignant lesions and their different grades. Although in the study carried out by Panneer Selvam *et al*. (15) a higher concentration is found in speckled leucoplakia, although not significantly. OPML patients have expressed higher levels of IL-6 compared to the CL group. Although Brailo *et al*. (25) have not found much higher levels. If we compare these patients with OSCC patients, it has been observed that the OSCC group expressed significantly higher concentrations of IL-6 except in the article by Juretić *et al*. (27) where there is no statistically significant difference.

Various studies (15,32) have compared salivary IL-6 concentrations between the different TNM stages of OSCC. As a result, Dineshkumar *et al*. (32) have not found significant differences in the different groups, unlike Panneer Selvam *et al*. (15) who have seen a statistically significant difference between S2 and S4, in favour of the most advanced stage.

**Figure 2:**
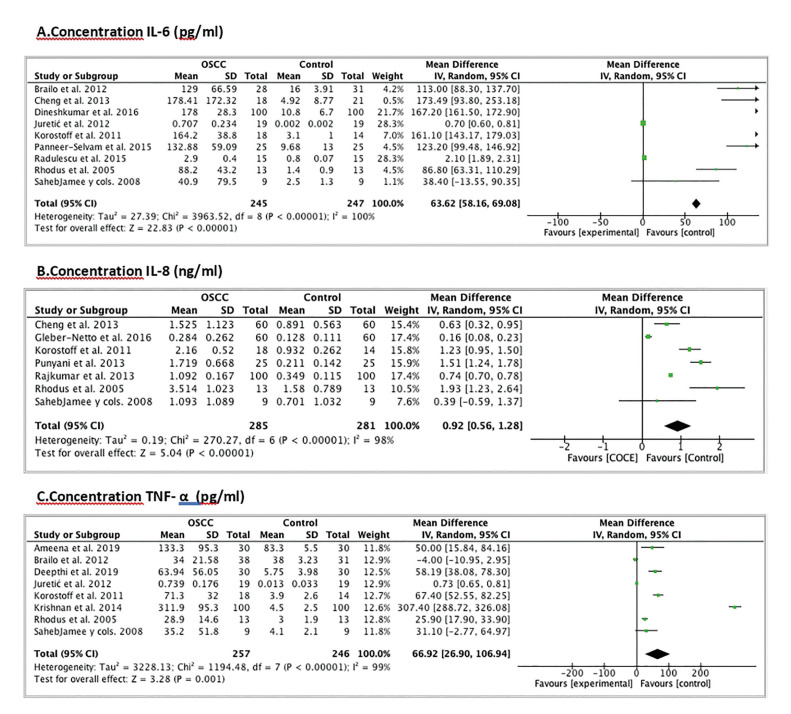
Forest Plot of the concentration differences between healthy patients and OSCC patients. A. IL-6 concentration (pg/ml); B. IL-8 concentration (ng/ml); C. TNF-α concentration (pg/ml).

Regarding the quantitative analysis, to study the differences in the concentration (pg/ml) of IL-6 we based ourselves on 9 articles (12,13,15,19,21,25,27,29,32). We discarded the study by St. John *et al*. (18) since it informs us of the concentration of IL-6 in serum and not in saliva; and we also rule out the studies by Katakura *et al*. (20) and Al Talabani *et al*. (22) since they do not provide us with the mean with the standard deviation of the IL-6 concentration. The population studied is 492 patients (245 from the OSCC group and 247 from the CL group). The difference in IL-6 concentration is statistically significant between the CL and OSCC group (Weighted Mean Difference (WMD): 63.62; 95% CI: 58.16 to 19.784, *p*<0.00001 and I2 heterogeneity: 100%, *p*<0.00001) (Fig. 2).

**Figure 3:**
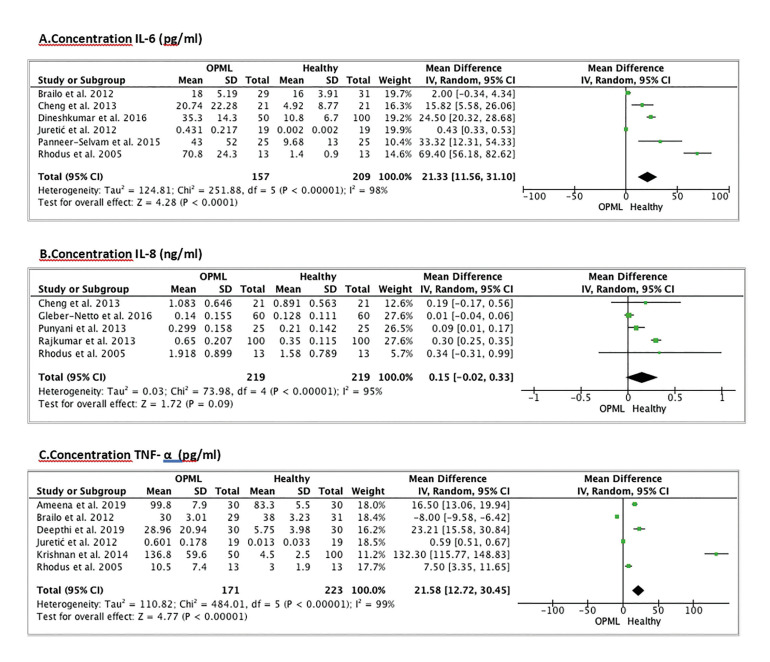
Forest Plot of the concentration differences between healthy patients and OPML patients. A. IL-6 concentration (pg/ml); B. IL-8 concentration (ng/ml); C. TNF-α concentration (pg/ml).

Meta-analysis is also performed to determine the differences in IL-6 concentration between the OPML group and the CL group. For this we choose 6 publications (13,15,19,25,27,32). The study by Khyani *et al*. (16) was excluded due to insufficient data being provided to perform the meta-analysis. The population is 366 patients (157 from the OPML group and 209 from the CL group). The difference in IL-6 concentration is statistically significant between both groups (Weighted Mean Difference (WMD): 21.33; 95% CI: 11.56 to 31.10, *p*<0.0001 and I2 heterogeneity: 98%, *p*<0.00001) (Fig. 3).

- IL-8

IL-8 has been evaluated in 14 publications (12,13,16,18-24,26,28,30,34) (Table 3) and in all of them it has been observed that this cytokine is present in higher salivary concentrations in OSCC group with respect to the OPML and CL groups. 7 of the studies (12,18,20-24) have compared only OSCC patients with CL patients, while 7 (13,16,19,26,28,30,34) have also compared salivary IL-8 levels in OSCC patients with OPML patients.

In most studies, it has been seen that there are statistically higher salivary levels of IL-8 in the OSCC group compared to the CL group, although in the studies carried out by Katakura *et al*. (20), SahebJamee *et al*. (21) and Korostoff *et al*. (12) no significant difference has been observed. In this last study, it has been seen that the endophytic type of OSCC did express statistically higher levels, while the exophytic type did not show significant differences with the CL group.

OPML have been leucoplakia, erythroplakia, erosive lichen planus and oral submucous fibrosis, with epithelial dysplasia (mild, moderate, and severe), and in one of the studies (13) a group of patients with chronic periodontitis (CP) has been analysed. In the study by Rajkumar *et al*. (28) and in that of Punyani *et al*. (26) no significant differences have been observed in the salivary levels of IL-8 between the different OPML and its different degrees. In the study by Lisa Cheng *et al*. (13) a higher concentration of IL-8 has been observed in patients with oral lichen planus compared to patients with CP. OPML patients have expressed higher levels of IL-8 compared to the CL group. Although Punyani *et al*. (27) and Gleber-Netto *et al*. (30) have not found much higher levels. The CP group has expressed a lower concentration of IL-8 than the CL group (13). If we compare these OPML patients with OSCC patients, it has been observed that the OSCC group expressed significantly higher concentrations of IL-8 in most of the studies, except for the one by Lisa Cheng *et al*. (13) where no statistically significant differences have been observed between the OSCC and OPML groups.

According to Singh *et al*. (34), in OSCC stages S3-S4, a higher concentration of IL-8 has been detected compared to S1-S2, and in the study by Brinkmann *et al*. (23) it has been observed that the most evolved primary tumors (T3-T4) express higher concentrations of IL-8 than T1-T2. In contrast, the studies by Punyani *et al*. (26) and Rajkumar *et al*. (28) have not observed statistically significant differences in the salivary levels of IL-8 between the different TNM stages of OSCC.

A meta-analysis is performed to assess the differences in concentration (ng/ml) of IL-8. For this we chose 7 studies (12,13,19,21,26,28,30). The studies by St. John *et al*. (18), Katakura *et al*. (20), Al Talabani *et al*. (22), Khyani *et al*. (16), Brinkmann *et al*. (23), Elashoff *et al*. (24) and that of Singh *et al*. (34) were excluded because they do not provide us with data to perform the meta-analysis. The population is 566 patients (285 from the OSCC group and 281 from the CL group). The difference in IL-8 concentration is statistically significant between the two groups (Weighted Mean Difference (WMD): 0.92; 95% CI: 0.56 to 1.28, *p*<0.00001 and I2 heterogeneity: 98%, *p*<0.00001) (Fig. 2).

A meta-analysis was also performed to determine the differences in IL-8 concentration between the OPML group and the CL group. For this we chose 5 publications (13,19,26,28,30). Studies by Khyani *et al*. (16) and Singh *et al*. (34) were excluded due to insufficient data being provided to perform the meta-analysis. The population is 438 patients (219 from the OPML group and 219 from the CL group). The difference in IL-8 concentration is not statistically significant between the two groups (Weighted Mean Difference (WMD): 0.15; 95% CI: -0.02 to 0.33, *p*=0.09 and I2 heterogeneity: 95%, *p*<0.00001) (Fig. 3).

- TNF-α

TNF-α has been evaluated in 9 publications (12,14,17,19,21,25,27,31,33) (Table 3). In 8 of the 9 studies (12,14,17,19,21,27,31,33) it has been observed that the OSCC group presents a higher concentration of TNF-α compared to the OPML and CL groups. SahebJamee *et al*. (21) have not found statistically significant differences in salivary concentration between the OSCC and CL groups, and only in the publication by Brailo *et al*. (25) show that the CL group presents a higher concentration compared to the OSCC and OPML group. 3 studies (12,21,31) have compared the salivary levels of OSCC patients with the CL group, while the rest (14,17,19,25,27,33) have also compared the OPML group.

OPML that have been observed are leucoplakia, oral submucous fibrosis, and erosive lichen planus, with epithelial dysplasia (mild, moderate, and severe). Deepthi *et al*. (33) is the only article that analyses and compares the concentrations of TNF-α in the different degrees of epithelial dysplasia, showing that the greater the severity of leucoplakia is, the higher the concentration of TNF-α is in saliva. OPML patients have expressed higher levels compared to the CL group, except for Brailo *et al*. (25) where their CL group has expressed higher levels of TNF-α. If we compare these patients with the OSCC patients, it has been observed that the OSCC group expressed a significantly higher concentration of TNF-α, although Brailo *et al*. (25) and Juretic *et al*. (27) did not find a statistically significant difference.

Krishnan *et al*. (14) and Ameena *et al*. (17) observed a higher salivary concentration of TNF-α in the more advanced OSCC stages, such as S3 and S4.

To evaluate the differences in concentration (pg/ml) of TNF-α, a meta-analysis was carried out based on 8 articles (12,14,17,19,21,25,27,33). The study by Polz-Dacewicz *et al*. (31) was excluded because they do not provide data to perform the meta-analysis. The population is 503 patients (257 from the OSCC group and 246 from the CL group). The difference in TNF-α concentration is statistically significant between the two groups (Weighted Mean Difference (WMD): 66.92; 95% CI: 26.90 to 106.94, *p*=0.001 and I2 heterogeneity: 99%, *p*<0.00001) (Fig. 2).

The meta-analysis allows us to determine the differences in TNF-α concentration between the OPML group and the CL group. For this we chose 6 publications (14,17,19,25,27,33). The population is 394 patients (171 from the OPML group and 223 from the CL group). The difference in TNF-α concentration is statistically significant between both groups (Weighted Mean Difference (WMD): 21.58; 95% CI: 12.72 to 30.45, *p*<0.00001 and I2 heterogeneity: 99%, *p*<0.00001) (Fig. 3).

## Discussion

Epidemiological data about the diagnosis of OSCC reveal the need to develop better tools to establish an early diagnosis of the malignant entity (2), and thus prevent its appearance in those patients at risk and improve the prognosis and survival of patients who have already developed the disease.

Saliva analysis is presented as a valid, reliable, cheap, easy, reproducible and, above all, non-invasive diagnostic tool, which could favour population screening and thus diagnose a multitude of nosological entities. In addition, due to these same characteristics, it presents multiple advantages when faced with blood or urine analysis, cell exfoliation and biopsy (5,7).

A multitude of studies have demonstrated the fundamental role of proinflammatory cytokines in the development of cancer (9,10). Although they have an important presence in the tumor microenvironment, these protein molecules are expressed in the patient's plasma, as well as in other biological fluids such as saliva and crevicular fluid (10). In this way, said fluids would be offering the opportunity to reveal and be able to detect early a local, regional, or systemic tumor process.

Despite the large number of salivary biomarkers that have been identified, there is a need to determine those that really have reliability and diagnostic utility (5,7). This systematic review focuses on the analysis of three proinflammatory cytokines (IL-6, IL-8 and TNFα) as potential salivary biomarkers with diagnostic capacity for OSCC. Other reviews have also analysed different cytokines such as IL-1β, IL-1α, IL-10, IL-1RA, IL-4, IL-13, IL-17; among other proinflammatory molecules (5,7,8).

The qualitative synthesis of the results (12-34) suggests that the three cytokines are expressed at higher concentrations in patients with OSCC compared with totally healthy patients and with OPML patients. A single study in the entire review has shown that the levels of one of the proinflammatory cytokines, TNF-α, have been lower in the OSCC group compared to the CL group (25). The systematic review and meta-analysis by Chiamulera *et al*. (5) follows the same pattern of results for IL-6 and IL-8, whereas TNF-α is not found in significantly elevated concentrations in OSCC patients compared to OPML patients.

These results suggest, as they do in the review by Ferrari *et al*. (7), that the three cytokines are closely associated with the carcinogenesis process and there is evidence that they could be potential OSCC biomarkers and be key in the differential diagnosis between a malignant lesion and a premalignant lesion (e.g., lichen planus, leucoplakia, erythroplakia, submucous fibrosis, different degrees of dysplasia). Furthermore, looking at the role of these proinflammatory cytokines in carcinogenesis, new therapeutic modalities for OSCC could be investigated. However, in the study by Chiamulera *et al*. (5) IL-6 and IL-8 were shown to be the most promising and reliable in the diagnosis of cancer, and the other cytokines tested (including TNF-α) did not show a significant difference in salivary concentration between OSCC patients and OPML patients.

Although it was not the main purpose of the review, in the studies that compared the salivary levels of any of the three cytokines between the different OPML, it has not been observed that any lesion expresses higher levels than the others. Although Panneer Selvam, *et al*. (15) has suggested that there are differences between the different types of leucoplakia. Ferrari *et al*. (7) find in 7 studies that the salivary levels of cytokines increase gradually when going from a well-differentiated lesion to a poorly differentiated lesion, and state that they are closely related to the aggressiveness and severity of the lesion. The latter is demonstrated in the study by Deepthi *et al*. (33), where it is observed that the more severe the leukoplakia, the higher the concentration of TNF-α is present in the saliva. It has also been possible to analyse in a secondary way the salivary concentration in the different TNM stages, and in some publications (8,15,23,26,28,32,34) a higher concentration has been observed against the higher the stage. Zielinska *et al*. (8) found that the salivary concentration of IL-17A, IL-17F and TNF-α was higher as the disease progressed and the stages increased. In addition, it was seen that, within the primary tumors, the T4 expressed higher values, as also demonstrated by Brinkmann *et al*. (23) with the salivary concentration of IL-8. Although in some studies there have been no statistically significant differences between the different stages, which can hinder and limit early detection of cancer (5,26,28,32).

Being able to develop a diagnostic test, based on the salivary concentration of any of the three cytokines analysed, that makes it possible to detect premalignant and malignant lesions in early stages, would be a favourable development for the prevention, prognosis, and survival of oral cancer, in addition to control and follow-up. in patients treated with OSCC. The dentist's attitude towards oral cancer should be aimed at preventing the disease (recognizing and controlling premalignant lesions and eliminating local chronic irritants), early diagnosis through examinations at periodic visits, providing adequate information to the patient (both in terms of prevention and of the disease itself), referral and reorientation of cancer patients to cancer treatment units and preventive dental treatment in those patients who are going to receive cancer treatment (3). For these reasons, this same group could play a fundamental role in its early detection using future reliable diagnostic tools (7).

Despite the extensive literature on this subject, future publications with an adequate methodology are needed to perform meta-analysis studies that determine reliable intervals for the salivary concentration of each biomarker. Regarding the limitations of this review, we found that several studies (16,18,20,22,23,24,31,34) lack the necessary data to perform their quantitative analysis. In addition, among the studies that have been quantitatively analysed, great variability has been found in the mean value of the concentrations of proinflammatory cytokines.

## Conclusions

Of the three interleukins considered in saliva, IL-6 and IL-8 have been the most studied and, in all studies, they have been expressed with higher salivary levels in patients with OSCC compared to those in the CL group and, in most, with compared to the OPML group. TNF-α has also been found at higher concentrations in the OSCC group compared to the CL and OPML group, except for a single study. The difference is statistically significant between the COCE group and the CL group. In contrast, the difference in the concentration of IL-6 and TNF-α is statistically significant between the LPMO group and the CL group, whereas it is not for IL-8.

In summary, this review highlights the importance of early detection in order to improve the prognosis of OSCC, and the three salivary cytokines have been shown to be a potential diagnostic tool.
